# Equidistant Intervals in Perspective Photographs and Paintings

**DOI:** 10.1177/2041669516662666

**Published:** 2016-08-17

**Authors:** Casper J. Erkelens

**Affiliations:** Department of Experimental Psychology, Helmholtz Institute, Utrecht University, Utrecht, The Netherlands

**Keywords:** bisection, equidistant intervals, visual space, physical space

## Abstract

Human vision is extremely sensitive to equidistance of spatial intervals in the frontal plane. Thresholds for spatial equidistance have been extensively measured in bisecting tasks. Despite the vast number of studies, the informational basis for equidistance perception is unknown. There are three possible sources of information for spatial equidistance in pictures, namely, distances in the picture plane, in physical space, and visual space. For each source, equidistant intervals were computed for perspective photographs of walls and canals. Intervals appear equidistant if equidistance is defined in visual space. Equidistance was further investigated in paintings of perspective scenes. In appraisals of the perspective skill of painters, emphasis has been on accurate use of vanishing points. The current study investigated the skill of painters to depict equidistant intervals. Depicted rows of equidistant columns, tiles, tapestries, or trees were analyzed in 30 paintings and engravings. Computational analysis shows that from the middle ages until now, artists either represented equidistance in physical space or in a visual space of very limited depth. Among the painters and engravers who depict equidistance in a highly nonveridical visual space are renowned experts of linear perspective.

## Equidistant Intervals in Pictures

**Figure 1.**
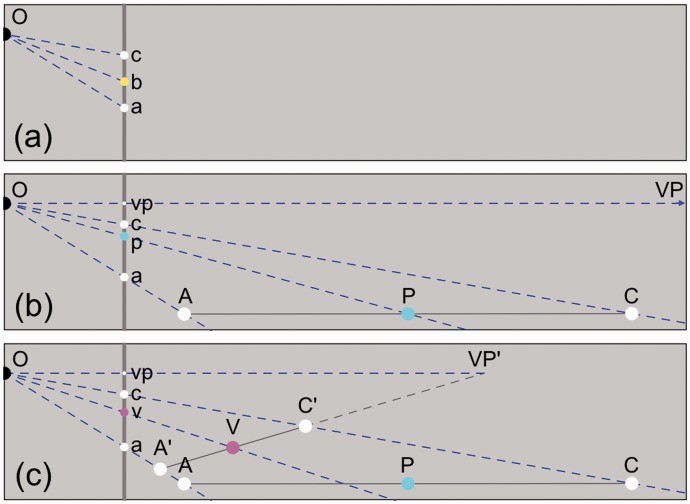
Bisection of a spatial interval in a picture in side views. Observer O views a picture (vertical gray line) that contains the proximal stimulus. (a) Bisection in the picture plane. The yellow dot b bisects interval ac if ab = bc. (b) Bisection in physical space. A, C, and VP are distal stimuli for a, c, and the vanishing point vp in the picture, respectively. AC is parallel to the line connecting O and the infinitely far VP. P bisects interval AC. The cyan dot p is the proximal stimulus of P. (c) Bisection in visual space. A, C, and VP are perceived at A′, C′, and VP′, respectively. VP′ is located at a finite distance. V bisects A′C′. The magenta dot v is the proximal stimulus of V.

Human vision is extremely sensitive to equidistance of spatial intervals in the frontal plane. Thresholds for spatial equidistance have been most extensively measured in bisecting tasks (Cappe, Clarke, Mohr, & Herzog, 2014; Chieffi, 1999; Fechner, 1860; Garcia-Suarez, Barrett, & Pacey, 2004; Levi & Klein, 1983; Milner, Brechmann, & Pagliarini, 1992; Nielsen, Intriligator, & Barton, 1999; van Vugt, Fransen, Creten, & Paquier, 2000; Varnava, McCarthy, & Beaumont, 2002; Volkmann, 1863; Westheimer, Crist, Gorski, & Gilbert, 2001; Wilkinson & Halligan, 2003). Despite the vast number of studies in normal subjects and patients, the neural mechanism behind visual equidistance is still largely unknown. Until now, research concentrated on bisection in the frontal plane. Little is known about the sensitivity of human vision to equidistance in depth. Even the spatial information employed still awaits discovery. Information may come from one of three possible sources. Figure 1 shows predictions for bisection of a spatial interval in a picture. Bisection may be related to the proximal (Figure 1(a)), distal (Figure 1(b)), or perceived (Figure 1(c)) stimulus. The image in the picture plane is taken to be equivalent with the proximal image. If information is extracted from the proximal stimulus, b will bisect interval ac if ab equals bc (Figure 1(a)). This type of bisection will be called proximal bisection. If bisection is related to the layout of the distal stimulus, p, that is, the projection of bisection point P of the physical interval AC onto the picture plane will generally not bisect interval ac (Figure 1(b)). The position of p is closer to c than a, if A is nearer to the observer than C. Lines parallel to AC meet at the infinitely far vanishing point VP. This type of bisection in a picture is dubbed bisection in physical space. A third option is that bisection is related to the layout of the stimulus in visual space (Figure 1(c)). Many studies have shown that visual space, that is, the space we perceive through vision, differs from physical space. A recent reanalysis of classic experimental results unveiled that physical space and visual space and also the proximal image are perspective transformations of each other (Erkelens, 2015a). The spaces differ by just a single parameter, namely, the distance of vanishing points. The distance is infinite for physical space, finite for visual space, and zero for the proximal image. The new model is simpler and more powerful than the model of a curved visual space championed by Luneburg (1947, 1950) and Blank (1953, 1961) because it describes more experimental data. In Figure 1(c), line piece AC in physical space transforms to line piece A′C′ in visual space. Lines parallel to AC in physical space converge in visual space to vanishing point VP′, located at finite distance from the observer. V bisects A′C′ in visual space. Point v is the projection of V onto the picture plane. The position of v differs from that of b (Figure 1(a)) and p (Figure 1(b)). Point v is located in between b and p at a position that depends on the distance of vanishing point VP′. In principle, human observers have access to each of the three sources of information. Proximal intervals are directly available from the retinal image. Most assessments of spatial intervals, however, are made across fixations and, hence, require a spatiotopic map of saccade locations in addition to the retinal image. Intervals in physical space follow from knowledge based on interactions of the observer with the physical environment. Intervals in visual space are available from quantitative probes of visual perception.

**Figure 2.**
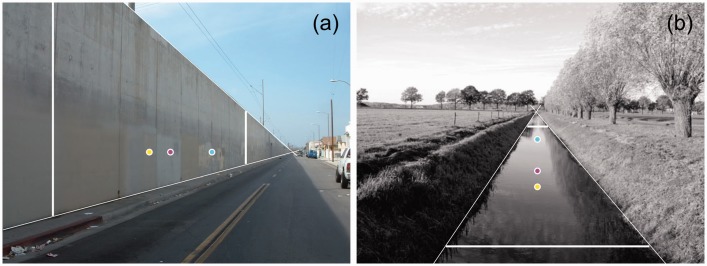
Computed bisection positions in photographs of perspective scenes. (a) Bisections along a vertical surface. The yellow dot bisects the interval between the parallel white lines in the picture. The cyan dot bisects the interval in physical space, the magenta dot in visual space. The end of visual space is assumed to be at twice the viewing distance. (b) Similar bisections along a horizontal surface. Converging white lines are perspective lines meeting at vanishing points.

To explore equidistance in perspective pictures, bisection locations were computed for spatial intervals in photographs of a wall and a canal (Figure 2). Pairs of parallel white lines represent spatial interval ac shown in Figure 1. Positions of the three bisection points were computed by solving the geometric equations that describe bisection according to the different sources of information. Measurement of the horizontal (wall) or vertical (canal) positions of the white lines in the photographs provided the positions of a and c. Positions of the vanishing point projections (vp) were obtained by measuring the positions of intersections of perspective lines in the pictures. The equations for bisection in the physical and visual spaces were solvable for known intersection positions between extensions of AC and A′C′ and the screen. A position at one screen size outside of the screen border was chosen as the position of intersection. Proximal bisection positions were directly computed from the positions of a and c on the screen. Veridicality of computed bisection positions in physical space was verified by counting the segments of the concrete wall and the trees along the canal in the picture. The positions of bisection in visual space were computed by further assuming that the vanishing point VP′ was located at twice the viewing distance of the photographs.

It requires a ruler to convince oneself that the yellow dots bisect the intervals between the parallel white lines in the photographs (Figure 2). The necessity of having to rely on a ruler suggests that equidistance judgments are not based on measures of the proximal stimulus. This suggestion is in line with earlier bisection results of Blank (1961) and a recent study showing that the detection of two-dimensional symmetry patterns is subject to the three-dimensional (3D) configuration of scenes (Chen & Shio, 2015). Acceptance of the cyan dots as indicators of bisection in physical space is based on cognition rather than visual perception. As mentioned earlier, counting trees and wall segments was needed to make educated guesses about bisection in physical space. Visually the cyan dots appear much too close to the distant lines. The magenta dots seem better candidates for visual bisection. Computed positions of the magenta dots depend on the assumed distance of vanishing point. Due to individual differences between computed depths of visual space (Erkelens, 2015b), the magenta dots may represent suboptimal positions for bisection to individual observers.

## Picture Positions of Equidistant Positions in Physical Space

**Figure 3.**
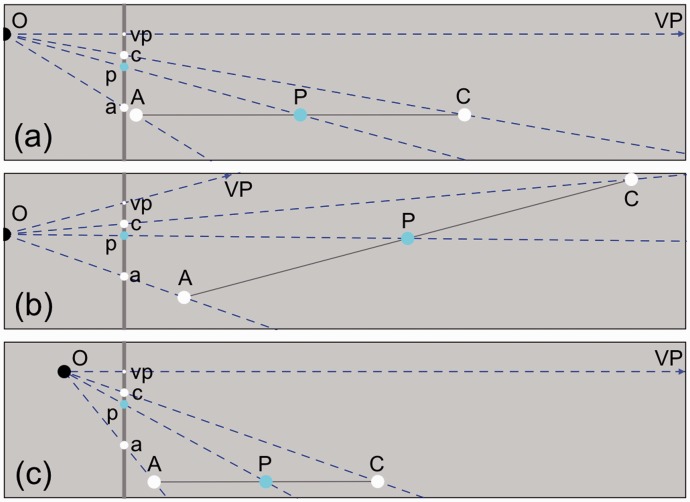
Independence of bisection in physical space for stimulus distance and viewing position. Points a, c, and vp have fixed positions in the picture (gray vertical line) identical to those shown in Figure 1(b). P bisects interval AC. p is the projection of P onto the picture plane. (a) In comparison to Figure 1(b), AC has been placed at a different distance from the screen. Different distances do not affect the position of p. (b, c) O has been placed at other positions. Different viewing directions do not affect p (b). Different viewing distances do not affect p (c).

To quantify the effect of the selected intersection positions between stimulus and screen on bisection positions in the picture, bisection positions were computed for a range of intersection positions. Bisection in physical space proved to be independent of the chosen intersection position. If a, c, and vp have fixed positions on the screen then all possible intervals AC in physical space are parallel to each other (Figure 1(b)). Inspection of Figures 1(b) and 3(a) shows that parallel displacement of AC does not affect the position of p because all triangles OAC are similar. On the other hand, bisection in visual space does depend on the position of the intersection point. Potential intervals A′C′ rotate relative to each other about the finite vanishing point and consequently the position of v changes with the orientation of A′C′. Bisection in physical space proved also to be independent of the position of the observer relative to the picture. Computations of p showed that if a, c, and vp were kept at fixed positions, the position of p remained fixed for all directions of O relative to the picture (Figure 3(b)). Computations of p also showed that the position of p remained fixed for all viewing distances (Figure 3(c)). As a result, the position of p in the picture is independent of distance and direction of the observer relative to the picture. The position of p is fully specified by the positions of a, c, and vp and thus only by aspects of the picture itself. Proximal bisection and bisection in physical space share this property.

**Figure 4.**
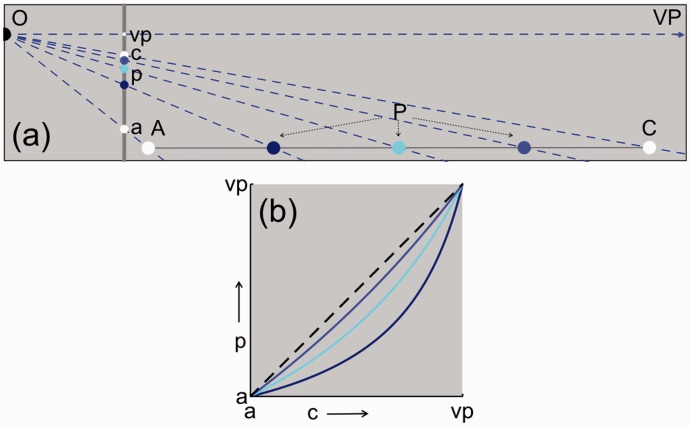
Equidistant intervals in physical space. Positions of a, c, and vp are identical to those shown in Figure 1(b). (a) AC is divided in four equidistant intervals. The colored dots P are associated with ¼, ½, and ¾ of interval AC, respectively. Identically colored p’s are their proximal stimuli. (b) The three p’s are computed as functions of c relative to a and v. Colors of the lines match the colors of P in (a).

The method for establishing bisection positions in pictures can be generalized to equidistant positions for any number of intervals. Computations were made for larger numbers of equidistant intervals. Figure 4(a) shows the geometry for a row of four equidistant intervals in physical space. Positions of the proximal stimuli p depend only on the positions of a, c, and vp. Figure 4(b) shows the positions of p as functions of the position of c relative to the positions of a and vp. Similar as for bisection, the positions of a, c, and vp in the picture uniquely specify the division in intervals. Geometric analysis shows that, what may seem counterintuitive, knowledge of the distal stimulus in physical space is irrelevant for the division of a line piece in equidistant intervals in a picture.

## Equidistant Intervals in Perspective Photographs

**Figure 5.**
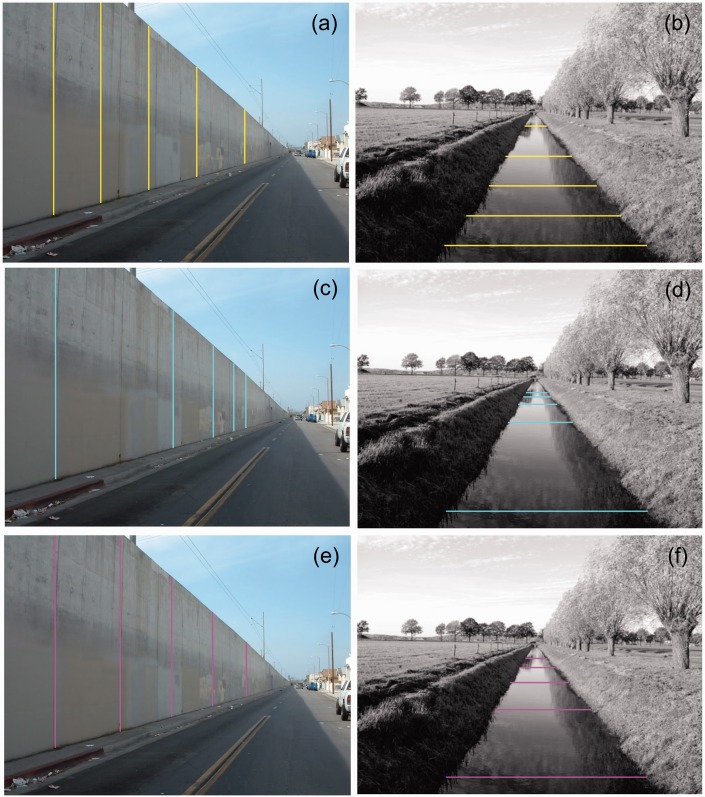
Equidistant intervals between five lines in perspective photographs of a wall and a canal. Intervals between yellow lines are equidistant in the picture plane (a, b). Intervals between cyan lines (c, d) are equidistant in physical space. Intervals between magenta lines (e, f) are equidistant in visual space. The vanishing point of visual space was assumed at twice the viewing distance of the pictures.

To compare equidistance in the various spaces, equidistant intervals were computed for the perspective photographs of wall and canal that were earlier shown in Figure 2. Figure 5 shows intervals that are equidistant in the picture (Figure 5(a) and (b)), in physical space (Figure 5(c) and (d)), and in visual space (Figure 5(e) and (f)). One can interpret the sizes of intervals in two ways. One can interpret their sizes in terms of distances in depth along the underlying surface or compare their sizes relative to each other in terms of viewing angles. Equidistant intervals in the plane of the picture (Figure 5(a) and (b)) are not perceived as equidistant according to either interpretation. Intervals that are identical in terms of visual angle but viewed at different distances are perceived differently according to the size—distance invariance hypothesis (Epstein, 1963; Wagner, 2006). The intervals are also perceived as different if one tries to ignore the depth of the surface. The perceived increase in line spacing with increasing surface depth even persists if a ruler is placed across the lines. Incompatibility between perceived intervals and ruler readings presents a strong visual illusion. Spatial intervals that are equidistant in physical space (Figure 5(c) and (d)) are also perceived as highly different from each other. By reasoning, one can accept that the intervals are equidistant along the receding surfaces. However, the decrease in line spacing appears to progress irregularly with surface depth. The near intervals seem overly large, most prominent in the canal example of Figure 5(d). Sizes of intervals appear to decrease more regularly with distance if the intervals are computed in visual space (Figure 5(e) and (f)). Similar to the computed bisection intervals of Figure 2, a distance of twice the viewing distance was chosen for the vanishing point. Now the intervals appear to decrease in size rather regularly with distance. Such a decrease is in agreement with the size—distance invariance hypothesis that was mentioned earlier. The gradient in interval size appears regular and more natural when the intervals are computed in visual (Figure 5(e) and (f)) rather than physical space (Figure 5(c) and (d)).

## Equidistant Intervals in Perspective Paintings and Engravings

To gain insight in how we perceive equidistance in pictures, it may be illuminating to investigate how painters, both amateurs and experts in linear perspective, have depicted equidistant intervals on canvas (Kemp, 1990; Kubovy, 1986). Veridical depiction of equidistant intervals in 3D scenes prescribes that painters have to follow the geometric rules of linear perspective in relation physical space. It is known that Italian painters such as Filippo Brunelleschi and Piero della Francesca already applied rules and constructions of linear perspective in the 15th century. To compare the depiction of equidistant intervals by artists across the ages, photographs of paintings and engravings were copied from the internet (see Appendix 1). Fragments were selected that contained four equidistant intervals and their associated vanishing point. An exception was made for “The Last Supper” and its followers because the images contained rows of only three equidistant intervals. The fragments were displayed on the computer screen so that they fully filled the screen. Coordinates of borders of intervals and vanishing points were measured in millimeters relative to the left and lower border of the screen, using a ruler. The near border of the nearest interval was designated a and the far border of the fourth (third for the Last Supper pictures) interval b (Figure 1). Intermediate interval borders were computed from the equations describing proximal equidistance, and equidistant intervals in physical and visual space, respectively. Since in visual space intervals depend on distance of the vanishing point and intersection position of the perceived surface with the screen, borders were computed for a wide range of vanishing distances and intersection positions. Combinations of vanishing distance and intersection position that produced the best fits between computed and painted borders were supposed to describe the visual space that was used by the painter.

**Figure 6.**
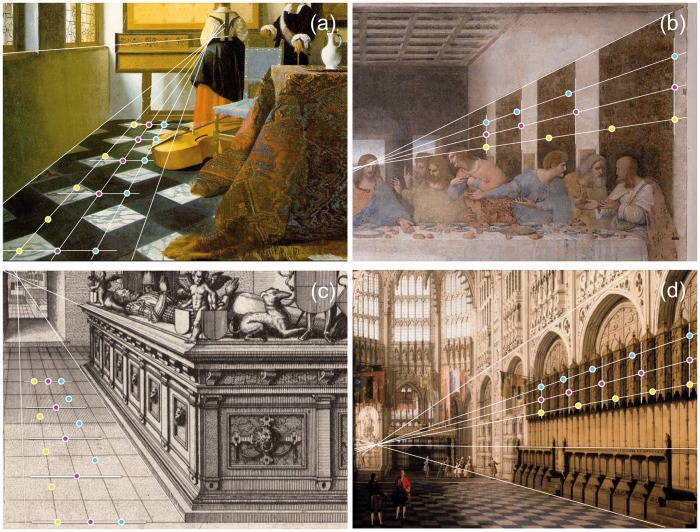
Equidistant intervals in art. (a) Fragment of “The Music Lesson”, painted by Johannes Vermeer. (b) Fragment of “The Last Supper”, painted by Leonardo da Vinci. (c) Fragment of a plate engraved by Hans Vredeman de Vries. (d) Fragment of “The Interior of Henry VII's Chapel in Westminster Abbey”, painted by Canaletto. Added converging white lines meet at vanishing points in the paintings. Added parallel white lines indicate equidistant intervals according to the artists. Cyan dots mark equidistant intervals in physical space. Magenta dots indicate equidistant intervals in visual space, and yellow dots are equidistant in the picture. Distances of the vanishing points in visual space are given in Tables 1 and 2.

Depth (D) of visual space in a picture is defined as distance of the vanishing point relative to the observer minus viewing distance of the picture. Figure 6 shows fragments of three paintings and one engraving. The tiles of Vermeer’s “The Music Lesson” show intervals that closely match equidistant intervals in a specific visual space (Figure 6(a)). The optimal visual space has a depth larger than 100 viewing distances. It is indistinguishable from physical space within the precision of the computations. Agreement of the painted intervals with intervals in physical space shows that Vermeer accurately followed the rules of linear perspective. It has been proposed that Vermeer used a camera obscura to achieve such a photograph-like veridicality (Steadman, 2001). The other panels of Figure 6 show that such an agreement was not always found in paintings and engravings. Leonardo da Vinci painted tapestries in “The Last Supper” that are not equidistant in physical space although the errors are not very large (Figure 6(b)). However, there is a visual space clearly different from physical space that describes Leonardo’s intervals much better. This visual space has a depth of 11 viewing distances. Differences between depicted and computed intervals in relation to physical space are much larger for the engraving of a tomb by Hans Vredeman de Vries (Figure 6(c)) and the painting of Canaletto of “The Interior of Henry VII's Chapel in Westminster Abbey” (Figure 6(d)). In both pictures, the best fitting borders that indicate equidistant intervals in visual space (magenta dots) are about in the middle between those indicating equidistant intervals in physical space (cyan dots) and the picture plane (yellow dots). Visual space in these pictures has a depth of just two viewing distances.

Depth of visual space was computed for 30 pictures. Paintings and engravings were selected in which perspective was applied with great accuracy. Paintings such as for instance “Mystery and Melancholy of a Street” (1914) of Giorgio de Chirico were excluded because of inaccuracy in vanishing points. Computed depths of visual spaces can be divided into two groups. In one group of 12 paintings and 2 engravings, depths are larger than 100 viewing distances (Table 1). These visual spaces are indistinguishable from physical space implying that the artists accurately applied the rules of linear perspective. In the other group of 15 paintings and 1 engraving, depths of visual spaces are shorter than 12 viewing distances (Table 2). Mean depth and standard deviation of this group is 5.2 ± 3.2 viewing distances. From the middle ages until now, artists have painted equidistant intervals in physical space (D > 100) or visual space. Mean absolute error (MAE) and standard deviation is 1.7 ± 1.0% for the physical space group and 1.4 ± 1.0% for the visual space group. This means that MAEs are usually just a few percent of interval lengths. Most artists applied exclusively perspective related to physical or visual space. The Dutch architect and painter Hans Vredeman de Vries (1527–1607) is here the exception. He applied the rules of linear perspective in relation to physical space with great accuracy in engravings, published in a book on perspective. On the other hand, he painted an architectural fantasy of a palace and engraved a scene with a tomb (Figure 6(c)) in a perspective space of just two viewing distances deep.

**Table 1.** List of Paintings and Engravings Containing Equidistant Intervals in Physical Space.

Artist	Title	Year	D	MAE
Fra Carnevale	Annunciation	1448	>100	0.7
Piero della Francesca	Madonna and Child with Saints	1472–1474	>100	1.8
Hans Vredeman de Vries	Plate 27 from the Book of Perspective	1604–1605	>100	0.4
Hans Vredeman de Vries	Plate 16 from the Book of Perspective	1604–1605	>100	1.4
Pieter Jansz. Saenredam	Interior St. Bavo Haarlem	1631	>100	1.9
Pieter de Hooch	A Woman Drinking with Two Men	1658	>100	2.8
Johannes Vermeer	The Music Lesson	1662–1664	>100	0.2
Johannes Vermeer	The Concert	1663–1666	>100	2.3
Gustave Caillebotte	Europe Bridge	1876	>100	1.2
Carel Willink	Simeon Styliticus	1939	>100	3.7
Carel Willink	Wilma with a Cat	1940	>100	2.9
Richard Estes	Williamsburg Bridge	2006	>100	1.0
Richard Estes	Supermarket Columbus Avenue	2009	>100	1.8
William R. Beebe	Columns in Capri	2010	>100	1.8
Means of 14 pictures			>100	1.7 ± 1.0%

*Note.* MAE = Mean absolute error. Computed depth (D) of visual space is expressed in viewing distances. Mean absolute error (MAE) between depicted and computed intervals is expressed as a percentage of interval lengths.

**Table 2.** List of Paintings and Engravings Containing Equidistant Intervals in Visual Space.

Artist	Title	Year	D	MAE
Ambrogio Lorenzetti	Annunciation	1344	4	1.4
Tommaso Masolino	Banquet of Herod	1435	6	2.5
Leonardo da Vinci	The Last Supper	1494–1499	11	0.5
Hans Vredeman de Vries	Architectural Caprice with Figures	1568	2	1.7
Hans Vredeman de Vries	Plate 16 from the book Pictures, etc.	∼1600	2	0.3
Dirck van Delen	Perspective Fantasy of a Palace with…	1650	3	0.8
Antonio Joli	Architectural Fantasy	1745	3	1.3
Giovanni Paolo Panini	Interior of the Santa Maria Maggiore	1750	8	0.6
Giovanni Battista Piranesi	Interior of the San Paolo fuori le Mura	1749	6	1.4
Canaletto	Capriccio, A Colonnade Opening	1765	4	3.0
Canaletto	Interior of Henry VII's Chapel	1770	2	0.2
Giacomo Raffaelli	The Last Supper	1816	12	0.5
Susan Dorothea White	The First Supper	1988	9	0.5
Roger Shepard	Terror Subterra illusion	1990	5	1.6
Primary School Arts Teacher	Perspective Aquariums	2012	3	2.7
Unknown	How to Draw Perspective (on YouTube)	2013	3	3.2
Means of 16 pictures			5.2 ± 3.2	1.4 ± 1.0%

*Note.* MAE = Mean absolute error. Computed depth (D) of visual space is expressed in viewing distances. Mean absolute error (MAE) between depicted and computed intervals is expressed as a percentage of interval lengths.

## Discussion

### Directions and Distances in Different Perspective Spaces

Recent analysis of classic experimental results provided evidence for the hypothesis that visual space is a perspective transformation of physical space (Erkelens, 2015a). Pictures as planar equivalents of proximal images are also perspective transformations of physical space. Physical space, visual space, and pictures are perspective spaces that just differ by the size of a single parameter, namely, the distance of vanishing points. Distances are infinite in physical space, finite in visual space, and zero in pictures. The idea of visual space as a perspective space arose from observations that perceived angles between in-depth oriented rails and bars are weighted averages between the physical angles and the angles in the proximal image (Erkelens, 2015b, 2015c). Perspective transformation of space affects distances but leaves directions unchanged. Therefore, vanishing points have identical directions relative to the observer in physical space, visual space, and the proximal image (Figure 1). As a consequence, studies on the quality of linear perspective in paintings and drawings are incomplete if analysis is confined to the accuracy of vanishing points. Complete studies of linear perspectives should include analysis of distances. The current study analyzed how equidistant intervals in physical and visual space relate to intervals in pictures.

The computations involved relative distances along straight lines. This means that linear errors in pictures caused by stretching did not affect the results. Linear errors occur for instance when changes are made in the aspect ratio of pictures. Higher order errors cause straight lines to become curved. Analysis of the vanishing points did not reveal such errors in photo’s and paintings that were downloaded from the internet.

One result of the current analysis is that, if intervals in a picture indicate equidistance in physical space, they do so for all viewing positions and distances of the physical stimulus to the picture. These invariances have not been reported until now and are a remarkable aspect of linear perspective in relation to physical space. The invariances do not hold for linear perspective in relation to visual space. Since the 15th century, many artists have used construction methods to depict intervals that are equidistant in physical space along in-depth oriented lines. A consequence of the invariances is that, if such methods are used, intervals in the picture still correctly indicate equidistant intervals in physical space for viewpoints other than the one from which the picture is made. The invariance may explain why photographs and movies viewed from oblique directions are perceived as being rotated rather than deformed (Cutting, 1987; Gombrich, 1972; Goldstein, 1979; Halloran, 1993; Koenderink, van Doorn, & Kappers 2004; Kubovy, 1986; Papathomas, Kourtzi, & Welchman, 2010).

### Equidistant Intervals in Paintings

It is remarkable how artists depict equidistant intervals in paintings and engravings. The studied intervals show that a great number of artists, intentionally or not, applied perspective in relation to visual instead of physical space. Among the painters who strongly deviated from equidistance in physical space are renowned experts of linear perspective such as Panini, Piranesi, and Canaletto (Thompson, 2003). The vanishing points in the paintings and engravings of this study show that all artists applied linear perspective with great accuracy, as far as directions are concerned. The small MAEs between depicted and computed intervals show that for distances painters applied perspective with great accuracy too. This is true for all artists in this study because, in general, MAEs were equally small for equidistance in visual and physical space. Small MAEs are remarkable for paintings showing equidistant intervals in visual space because their makers could not use tools or tricks to achieve a high degree of perfection. In contrast, artists drawing equidistant intervals in physical space could use methods such as the costruzione legittima. Such methods do not exist for equidistance in visual space. Making use of yardsticks in the plane of the picture would not have been helpful either, as the yellow dots show in Figure 6.

Artists have pictured equidistant intervals either in physical or visual space from the 15th century until now. Realist painters such as Johannes Vermeer, Gustave Caillebotte, Carel Willink, Richard Estes applied perspective in relation to physical space to arrive at photograph-like veridicality. Painters such as Leonardo da Vinci, Hans Vredeman de Vries, and Dirck van Delen used perspective in relation to visual space in their fantasy paintings. The vedute painters Antonio Joli, Giovanni Panini, Giovanni Piranesi, and Canaletto applied perspective in relation to visual space both in their fantasy and realistic paintings. From visual inspection alone, it is difficult to distinguish nonveridical from veridical perspective. For example, “The Last Supper” and the “tomb” of Hans Vredeman de Vries appear as realistic as “The Music Lesson” and the “Interior of Henry VII's Chapel” (Figure 6). Computation first shows the considerable differences. Hans Vredeman de Vries, an engineer by training, may have been aware of the distinction between both types of perspective because he applied both techniques. He purposely may have applied perspective in relation to visual space for one or two reasons. He may have found the painting more attractive or he used it because it allowed him to draw distant objects in more detail than in the case of linear perspective. Distant objects are most compressed in linear perspective, less in visual space, and not at all in physical space. The latter reason is particularly relevant for engravings where depicting details is limited by the coarseness of the needles. It is amusing to notice that perspective in relation to visual space has been applied in the “Terror Subterra” illusion (Shepard, 1990). It is amusing because the illusion is generally explained by the size–depth relationship of projections from physical space onto planar surfaces. Another example of erroneous projection is given by an instruction video on how to draw perspective (last item of Table 2). It shows intervals that are equidistant within an extremely confined visual space. Apparently, both viewers and makers are not aware of the errors of projection.

The fact that expert painters depict perspective in relation to visual space seems paradoxical. Projections of 3D scenes on flat surfaces contain linear perspective. These photographic projections produce retinal images identical to those produced by the 3D scenes themselves. When using projections based on perspective in relation to visual space in the painting, one would be inclined to conclude that the ensuing visual perception of the scene would be nonveridical and nonrealistic. On the contrary, perspective related to visual space is experienced as very natural in paintings (see the examples in Figures 5 and 6). An explanation for the paradox may be that perception of 3D scenes in pictures differs from perception of real scenes (Erkelens, 2015b, 2015c). To emphasize this difference, visual space in pictures is generally named pictorial space. Apparently, about half of the investigated painters used perspective in relation to visual space so that in the pictures the depicted intervals appeared consistent with other attributes of depth.

## Appendix 1

### URLs of the Pictures of Table 1

Fra Carnevale, http://www.wga.hu/index1.html

Piero della Francesca, http://www.wga.hu/index1.html

Hans Vredeman de Vries, www.relewis.com/devries-perspective_link.html

Hans Vredeman de Vries, https://ojs.st-andrews.ac.uk/index.php/nsr/article/download/748/921

Pieter Jansz. Saenredam, https://commons.wikimedia.org/wiki/File:Pieter_Jansz._Saenredam,_Dutch_(active_Haarlem_and_Utrecht)_-_Interior_of_Saint_Bavo,_Haarlem_-_Google_Art_Project.jpg

Pieter de Hooch, https://en.wikipedia.org/wiki/A_Woman_Drinking_with_Two_Men#/media/File:Pieter_de_Hooch_-_A_Woman_Drinking_with_Two_Men_-_WGA11694.jpg

Johannes Vermeer, https://en.wikipedia.org/wiki/The_Music_Lesson#/media/File:Jan_Vermeer_van_Delft_014.jpg

Johannes Vermeer, https://upload.wikimedia.org/wikipedia/commons/c/c8/Vermeer_The_concert.JPG

Gustave Caillebotte, https://upload.wikimedia.org/wikipedia/commons/0/00/Caillebotte-PontdeL%27Europe-Geneva.jpg

Carel Willink, http://cultured.com/images/image_files/2864/3597_o_simeon_styliticus.jpg

Carel Willink, http://www.wikiart.org/en/carel-willink/wilma-with-a-cat-1940

Richard Estes, http://www.phaidon.com/resource/estes-099.jpg

Richard Estes, http://www.artnet.com/usernet/awc/awc_workdetail.asp?aid=139829&gid=139829&cid=17191&wid=425966615&page=3

William R. Beebe, http://www.williamrbeebe.com/european-landscapes/columns-in-capri

### URLs of the Pictures of Table 2

Ambrogio Lorenzetti, https://en.wikipedia.org/wiki/Annunciation_(Ambrogio_Lorenzetti)

Tommaso Masolino, https://upload.wikimedia.org/wikipedia/commons/3/34/Masolino_-_Banquet_of_Herod_-_WGA14245.jpg

Leonardo da Vinci, https://upload.wikimedia.org/wikipedia/commons/4/4b/%C3%9Altima_Cena_-_Da_Vinci_5.jpg

Hans Vredeman de Vries, http://digi.ub.uni-heidelberg.de/diglit/vries1620/0016

Hans Vredeman de Vries, https://upload.wikimedia.org/wikipedia/commons/f/f0/Hans_Vredeman_de_Vries_-_Architectural_Caprice_with_Figures_-_Google_Art_Project.jpg

Dirck van Delen, http://artuk.org/discover/artworks/perspective-fantasy-of-a-palace-with-elegant-figures-99902

Antonio Joli, http://www.artchive.com/web_gallery/A/Antonio-Joli/Architectural-Fantasy.html

Giovanni Paolo Panini, http://www.wga.hu/html_m/p/pannini/

Giovanni Battista Piranesi, https://commons.wikimedia.org/wiki/File:Giovanni_Battista_Piranesi,_St._Paolo_fuori_le_Mura_Interior.png

Canaletto, http://www.histoiredelart.net/core/class/getimage.php?img=artiste/777/1352287367.jpg

Canaletto, http://www.sothebys.com/en/auctions/ecatalogue/2016/master-paintings-evening-sale-n09460/lot.59.html

Giacomo Raffaelli, http://vopblog.com/wp-content/uploads/2015/10/ThinkstockPhotos-160585076.jpg

Susan Dorothea White, http://www.susandwhite.com.au/enlarge.php?workID=94

Roger Shepard, http://www.anopticalillusion.com/2015/09/terror-subterra-by-roger-shepard/

Primary School Arts Teacher, http://www.onceuponanartroom.com/2012_03_01_archive.html

Unknown, https://www.youtube.com/watch?v=phWtQ2odZh0
